# Tourniquet‐induced lower limb ischemia/reperfusion reduces mitochondrial function by decreasing mitochondrial biogenesis in acute kidney injury in mice

**DOI:** 10.14814/phy2.15181

**Published:** 2022-02-10

**Authors:** Balamurugan Packialakshmi, Ian J. Stewart, David M. Burmeister, Yuanyi Feng, Dennis P. McDaniel, Kevin K. Chung, Xiaoming Zhou

**Affiliations:** ^1^ Department of Medicine Uniformed Services University of the Health Sciences Bethesda Maryland USA; ^2^ The Henry Jackson M. Foundation for the Advancement of Military Medicine Bethesda Maryland USA; ^3^ Department of Biochemistry Uniformed Services University of the Health Sciences Bethesda Maryland USA; ^4^ Biomedical Instrumentation Center Uniformed Services University of the Health Sciences Bethesda Maryland USA

**Keywords:** autophagy, ischemia, mitochondrial complex, mitochondrial oxidative stress, mitophagy

## Abstract

The mechanisms by which lower limb ischemia/reperfusion induces acute kidney injury (AKI) remain largely uncharacterized. We hypothesized that tourniquet‐induced lower limb ischemia/reperfusion (TILLIR) would inhibit mitochondrial function in the renal cortex. We used a murine model to show that TILLIR of the high thigh regions inflicted time‐dependent AKI as determined by renal function and histology. This effect was associated with decreased activities of mitochondrial complexes I, II, V and citrate synthase in the kidney cortex. Moreover, TILLIR reduced mRNA levels of a master regulator of mitochondrial biogenesis PGC‐1α, and its downstream genes NDUFS1 and ATP5o in the renal cortex. TILLIR also increased serum corticosterone concentrations. TILLIR did not significantly affect protein levels of the critical regulators of mitophagy PINK1 and PARK2, mitochondrial transport proteins Tom20 and Tom70, or heat‐shock protein 27. TILLIR had no significant effect on mitochondrial oxidative stress as determined by mitochondrial ability to generate reactive oxygen species, protein carbonylation, or protein levels of MnSOD and peroxiredoxin1. However, TILLIR inhibited classic autophagic flux by increasing p62 protein abundance and preventing the conversion of LC3‐I to LC3‐II. TILLIR increased phosphorylation of cytosolic and mitochondrial ERK1/2 and mitochondrial AKT1, as well as mitochondrial SGK1 activity. In conclusion, lower limb ischemia/reperfusion induces distal AKI by inhibiting mitochondrial function through reducing mitochondrial biogenesis. This AKI occurs without significantly affecting PINK1‐PARK2‐mediated mitophagy or mitochondrial oxidative stress in the kidney cortex.

## Graphical abstract

Lower limb ischemia/reperfusion induces distal AKI by inhibiting mitochondrial function through reducing mitochondrial biogenesis. This AKI occurs without significantly affecting PINK1‐PARK2‐mediated mitophagy or mitochondrial oxidative stress in the mouse kidney cortex.

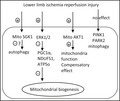

## 1 INTRODUCTION

Limb ischemia/reperfusion (I/R) from various types of injury, disease states, and surgical procedures (e.g. thromboembolic events and vascular surgery on the extremities) can induce metabolic disturbances that lead to distant organ damage such as acute kidney injury (AKI) (Arora et al., 2015; Chavez et al., 2016; De Rosa et al., 2018; Gallo et al., 2021; Kasepalu et al., 2020; Leurcharusmee et al., 2018; Morsey et al., 2003). Ineffective management of AKI can result in death (Heegard et al., 2015; Wohlauer et al.,). Tourniquet‐induced lower limb I/R (TILLIR) AKI may also occur in the military operational setting from the use of limb or junctional tourniquets, or the use of other hemostatic maneuvers such as resuscitative endovascular balloon occlusion (Hoareau et al., 2019; Kragh et al., 2008; Muñoz et al., 2020). I/R of any large muscle mass may induce rhabdomyolysis with the release of myoglobin, danger‐associated molecular patterns and other inflammatory cytokines. The resulting systemic inflammation increases vascular permeability, leading to intravascular volume depletion followed by vasoconstriction via activation of the renin‐angiotensin‐aldosterone pathway and consequent reduction of renal blood flow and oxygenation. Furthermore, elevated circulating myoglobin may lead to occlusion of glomerular arterioles and cast formation in the renal tubules that have direct cytotoxic effects on tubular cells (De Rosa et al., 2018; Simon et al., 2018). Thus, the kidney after TILLIR may be chronically hypoperfused and hypoxic. This is in contrast to AKI caused by surgery (e.g., cardiac/thoracic/abdominal surgery), renal transplant, and trauma in which the blood supply to the kidney is compromised for shorter periods of time with relatively rapid restoration of normal or near normal renal perfusion.

The health of renal mitochondria is an important factor for both the pathogenesis of and recovery from AKI (Forbes, 2016). Mitochondria are dynamic organelles homeostatically balanced by mitochondrial biogenesis and mitophagy. Mitochondrial biogenesis requires coordinated signaling amongst cytosolic, nuclear, and mitochondrial compartments. Peroxisome proliferator‐activated receptor‐γ coactivator‐1α (PGC‐1α) orchestrates much of this signaling and is regarded as a master regulator of mitochondrial biogenesis (Clark & Parikh, 2021). Although reduced expression of PGC‐1α has been implicated in AKI secondary to endotoxemia (Tran et al., 2011), folic acid (Fontecha‐Barriuso et al., 2019; Ruiz‐Andres et al., 2016), experimental renal artery stenosis (Farahani et al., 2020), renal pedicle‐clamping (Collier & Schnellmann, 2020; Song et al., 2021), cisplatin (Portilla et al., 2002), and pathophysiologic levels of glucocorticoids (Annie et al., 2019; Rahnert et al., 2016), its role in TILLIR remains to be elucidated.

Autophagy serves a defense mechanism maintaining normal cellular homeostasis via recycling of intracellular contents (Dodson et al., 2013; Suliman et al., 2021; Wang, Zhu, Li, Ren, & Zhou, 2020). Experimental rhabdomyolysis, cisplatin, and renal I/R‐induced AKI increases autophagy as a protective mechanism (Funk & Schnellmann, 2012; Jiang et al., 2012; Kimura et al., 2011), whereas lipopolysaccharide‐induced sepsis inhibits autophagy, which contributes to renal injury (Zhao et al., 2020). Autophagy begins with formation of the phagophore and concludes with degradation of autophagosomes by lysosomal hydrolases (Dodson et al., 2013). Classic autophagic flux involves conversion of cytosolic protein LC3‐I to LC3‐II, leading to recruitment of LC3‐II and p62/SQSTEM1‐attached cargoes to the membrane of autophagosomes (Dodson et al., 2013; Suliman et al., 2021; Wang, Zhu, Li, Ren, & Zhou, 2020). Mitophagy is a mechanism for removing damaged mitochondria through autophagic machinery to control mitochondrial generation of reactive oxygen species (Wang, Zhu, Li, Ren, Zhang, et al., 2020; Wang, Zhu, Toan, et al., 2020). The PTEN‐induced putative kinase 1 (PINK1)‐parkin RBR E3 ubiquitin protein ligase (PARK2) pathway is the best‐characterized regulatory mechanism for mitophagy. Sepsis and renal‐I/R‐induced AKI activates mitophagy by increasing PINK1 and PARK2 protein levels (Tang et al., 2018; Wang, Wang, et al., 2020; Wang et al., 2021), whereas genetic ablation of PINK1 and/or PARK2 exacerbates renal I/R or sepsis‐induced AKI (Tang et al., 2018; Wang et al., 2021). These data indicate that PINK1 and PARK2‐mediated mitophagy is a protective mechanism against sepsis and renal I/R‐induced AKI. Likewise, the beneficial effects of ischemia preconditioning and annexin A1 tripeptide against renal I/R‐induced AKI (Suliman et al., 2021; Wang, Zhu, Li, Ren, & Zhou, 2020) and heat shock protein‐27 (HSP‐27) against cardiac cell death (Kang et al., 2011; Lin et al., 2016) are mediated in part by PINK1‐PARK2‐dependent mitophagy.

Serine/threonine kinases including extracellular signal‐regulated kinases 1 and 2 (ERK1/2), AKT1, and serum/glucocorticoid‐stimulated kinase 1 (SGK1) all help to regulate mitochondrial function, biogenesis, autophagy and mitophagy in the kidney and other organs (Larson‐Casey et al., 2016; Park et al., 2017; Soutar et al., 2018). For example, in renal I/R or oxidative stress‐induced renal cell injury, ERK1/2 inhibits mitochondrial function through decreased expression of PGC‐1α (Collier & Schnellmann, 2020; Nowak et al., 2006).

Despite the grave consequences I/R‐induced AKI may impose, little is known about the pathophysiology of TILLIR‐induced AKI. We first sought to determine the effect of TILLIR on mitochondrial function in the renal cortex due to the high energy demands there (Bhargava & Schnellmann, 2017). We found that TILLIR caused significant AKI and decreased renal mitochondrial function. This effect on mitochondrial function was explained by decreased mitochondrial content through reducing mitochondrial biogenesis without significantly affecting PINK1‐PARK2‐mediated mitophagy or mitochondrial reactive oxygen species. Furthermore, in contrast to direct I/R of the kidney, TILLIR reduced autophagy. Having found that the effect of TILLIR‐induced AKI on autophagy and mitophagy is somewhat different from the effect of other types of AKI, we examined the effects of TILLIR on ERK1/2, AKT1 and SGK1. TILLIR increased phosphorylation (activation) of both cytosolic and mitochondrial ERK1/2, but only activated mitochondrial AKT1 and SGK1. Thus, we have discovered a novel mechanism by which lower limb I/R induces distal AKI.

## 2 METHODS

### 2.1 Mice

**FIGURE 1:**
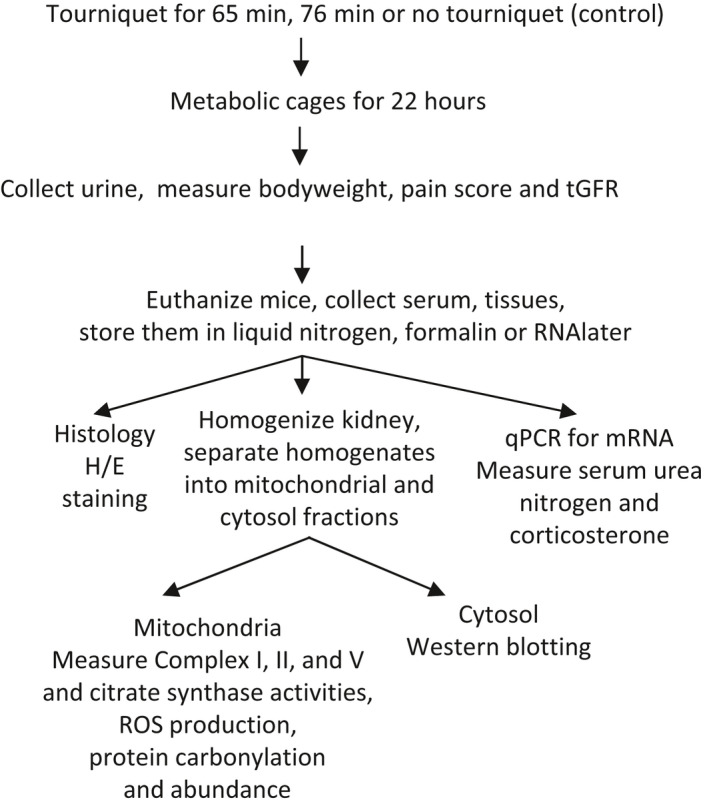
Summary of experiment procedures

Use of mice was approved by the institutional animal care and use committee of the Uniformed Services University of the Health Sciences. Male C57BL/6 mice (5–8 weeks old) were chosen because male mice are more susceptible to develop AKI than females (Park et al., 2004). Furthermore, direct renal I/R injury is twice as lethal in men as in women (Kher et al., 2005). Mice were weighed and anesthetized with ketamine and xylazine. TILLIR was induced by placing bilateral latex‐O‐rings (Orthodontic Elastics, 3.2 mm, heavy force 4.5 oz) on the inguinal regions of the mice for either 65 or 76 min at 30°C. After releasing tourniquets, mice were placed individually in metabolic cages to collect urine for 22 h. Control mice were treated with the same procedures but without Latex‐O‐rings. At the end of 22 h, mice were again weighed, and scored for pain as defined by hind limb mobility, appearance and provoked responses (Koch et al., 2016), before additional experiments were performed. The procedures are summarized in Figure 1.

### 2.2 Transcutaneous glomerular filtration rate (tGFR) measurement

tGFR was measured transdermally with the clearance of FITC‐sinistrin, as described previously (Scarfe et al., 2018). Briefly, under isoflurane anesthesia, mice were injected with FITC‐sinistrin via the intraorbital route and the disappearance of the FITC fluorescence was monitored by a detector mounted on the shaved lumbar region of mice. The half‐time of reduction in the blood sinistrin concentrations is proportional to GFR.

### 2.3 Serum urea nitrogen (SUN) measurement

After tGFR measurements, blood was drawn via cardiac puncture. SUN levels were detected with the diacetyl monoxime method, as described previously (Rahmatullah & Boyde, 1980). Briefly, a chromogen was freshly prepared by mixing Reagent A (10 mg of ferric chloride dissolved in 10 ml of 1.48 M phosphoric acid mixed with 30 ml of 5.52 M sulfuric acid and 60 ml of distilled water) and Reagent B (50 mg of diacetyl monoxime and 1 mg of thiosmicarbazide dissolved in 10 ml of water) at a 2:1 ratio. Serum samples were diluted 10 times with de‐ionized water, and proteins were precipitated with 0.61 M trichloroacetic acid solution and centrifuged at 10,000*g* for 10 min at room temperature. The resulting supernatants were incubated with the chromogen at 80°C for 5 min, and absorption was measured at 490 nm.

### 2.4 Histology

Mice were first perfused retroventrically with ice‐cold PBS and then with 10% formalin. The kidney was embedded, sectioned at 4 µm, and stained with hematoxylin/eosin. Slides were then scanned with a Zeiss Axioscan Z1 slide scanner (Carl Zeiss).

### 2.5 Immunohistochemistry

The assay was performed as previously described (Houlihan et al.,). Briefly, paraffin‐embedded kidney slides were deparaffinized and rehydrated with xylene, ethanol and water sequentially. Antigens were unmasked by incubating slides in an Antigen unmasking solution (H3300, Vector lab) and boiling for 20 min. Non‐specific binding was blocked by incubation with 0.05% Triton‐X 100 and 5% normal goat serum (005‐000‐121, Jackson ImmunoResearch) in PBS at room temperature for 1 h. Slides were then incubated with a rabbit antibody against cleaved caspase‐3 (9661, Cell Signaling Technology) and mouse antibody against β‐catenin (610153, BD Transduction Laboratories) and Hoechst 33342 for staining nuclei (Molecular Probes) at 4°C overnight. An Alexa Fluor 488 AffiniPure Goat ant‐rabbit IgG (111‐545‐144, Jackson ImmunoResearch) and a Cy3 AffiniPure Goat anti‐mouse IgG (115‐165‐146, Jackson ImmunoResearch) were used to recognize the respective anti‐cleaved caspase‐3 and β‐catenin antibodies.

### 2.6 Transmission electron microscopy

After euthanasia, the kidney cortex identified by anatomic location was cut immediately into 1 mm^3^ blocks, which were fixed in 2% formaldehyde, 2% glutaraldehyde, and 1% tannic acid in PBS (pH 7.2) overnight at 4°C followed by three 15 min washes in PBS at room temperature. Samples were incubated in cacodylate buffer (CB, 0.2 M, pH 7.2) for 15 min for three times to remove phosphate ions that cause precipitation in the presence of osmium. Samples were then post‐fixed in 2% OsO_4_ in CB for 1 h at room temperature followed by three times 10 min washes in CB. Samples were dehydrated in a graduated series of ethanol, infiltrated with Spurr's epoxy resin (Electron Microscopy Sciences) and polymerized at 70°C for 12 h. Following polymerization, ultrathin sections (70–80 nm) were cut on a Leica UC6 ultramicrotome (Leica Microsystems) and collected on 200‐mesh copper grids. Grids were post‐stained in 2% aqueous uranyl acetate for 15 min and Reynold's lead citrate for 5 min and examined on a JEOL JEM‐1011 transmission electron microscope (JEOL USA, Inc.). Images were captured on an Advanced Microscopy Techniques 4MP digital camera (AMT Corp). The mitochondrial length was measured with the software AmtV602 Image Capture Engine. Data are expressed as percentages of tubular cells that have <1% of mitochondria longer than 2 μm (Brooks et al., 2009). A total of 30 cells were counted in each group.

### 2.7 Isolation of mitochondria, mitochondrial function, citrate synthase activity, and serum corticosterone assays

Mitochondria were isolated as previously described (Hira et al., 2019; Packialakshmi & Zhou, 2018). Briefly, the kidney cortex in a ratio of 5 µl of IB buffer [225 mM mannitol, 75 mM sucrose, 0.1 mM EGTA, 30 mM Tris‐HCl and protease inhibitor tablet (Roche), pH 7.5] per mg of tissue was homogenized with an electrically powered motor (Wheaton Overhead Stirrer) for 40 s. The homogenate was centrifuged at 4°C at 600*g* for 20 min. The pellet was discarded, while the supernatant was collected and centrifuged again at 4°C at 10,000*g* for 10 min. After this centrifugation, the cytosolic fraction (supernatant) was collected and the mitochondrial fraction (pellet) was washed once with the same buffer and centrifuged again at 4°C at 10,000*g* for 10 min. The pellet was then suspended in IB buffer. A BCA assay was used to determine the protein concentrations of both cytosolic and mitochondrial extracts.

Activities of the mitochondrial complex I and complex II were measured as previously described (Packialakshmi & Zhou, 2018). Briefly, for measuring complex I activity, mitochondrial extracts (5 μg/well) were incubated with 25 mM of potassium phosphate, 3.5 g/L of BSA, 60 μM of 2,6‐dichloroindophenol, 70 μM of decylubiquinone, 1 μM of antimycine A, 0.2 mM of NADH, and 1 μM of rotenone in a 96‐well plate at room temperature to generate superoxide, which oxidizes 2,6‐dichloroindophenol, resulting in decreases at A_620_. The difference in A_620_ with and without mitochondria reflects the activity of complex I. For measuring complex II activity, mitochondrial extracts (5 μg/well) were incubated with 80 mM of potassium phosphate, 1 g/L of BSA, 2 mM of EDTA, 0.2 of mM ATP, 80 μM of 2,6‐dichloroindophenol, 50 μM of decylubiquinone, 1 μM of antimycine A, 1 μM of rotenone, 10 mM of succinate, and 2 mM of KCN. The difference in A_620_ with and without mitochondria reflects the activity of complex II. The standard curves for complex I and II were generated with different concentrations of 2,6‐dichloroindophenol in the absence of mitochondria. Complex V (F0F1‐ATPase) activity was measured as described by Law et al (Law et al., 1995), but released inorganic phosphate was measured by Melachite green buffer from Millipore (Zhou et al., 2017). Mitochondrial citrate synthase and serum corticosterone activities were measured with kits from Cayman Chemical with item #701040 and #501320, respectively, according to the manufacturer's protocols.

### 2.8 Mitochondrial oxidative stress assay

Mitochondrial ability to generate reactive oxygen species was measured as previously described (Packialakshmi & Zhou, 2018). Briefly, mitochondrial extracts (5 μg/well) were incubated in 50 mM of K_3_PO_4_ pH 7.8, 1 mM of DETAPAC, 1 unit of catalase, 0.5 mg/ml of salmon sperm DNA, 80 μM of antimycin A, 5 mM of succinate, 10 μM of dihydroethidium in a black 96‐well viewplate (PerkinElmer) at 37°C for 30 min. Fluorescence was measured with excitation at 485 nm and emission at 610 nm. The total mitochondrial protein carbonylation was measured using a fluorescence method with a kit from Cayman Chemical according to the manufacturer's protocol (Catalog # 701530). Carbonylation of specific mitochondrial proteins was measured with a kit form Cell Biolabs (Catalog# STA‐308).

### 2.9 Western analysis

Both cytosolic and mitochondrial samples were dissolved in SDS loading buffer with addition of 2 mM of NaF and 2 mM of Na_3_VO_4_ to inhibit phosphatases. The mitochondrial fractions were also sonicated for 5 s to break mitochondrial DNA and facilitate loading. Samples were loaded at 20–30 μg protein/lane and separated on 4%–12% Bis‐Tris gels (ThermoFisher, Catalog# NP0336BOX or NW04127BOX). Proteins were transferred to a nitrocellulose membrane (ThermoFisher, Catalog# LC2001). After transferring, the gel was stained with SimplyBlue^tm^ SafeStain (ThermoFisher, Catalog # LC6060) to inspect sample loadings. The membrane was blocked with a blocking buffer (Odyssey, Part # 927‐40000) for 1 h at room temperature. The membrane was probed with a primary antibody generally at 1:1000 dilution at 4°C overnight. The membrane was then washed briefly and probed with an Alexa fluorophore conjugated secondary antibody at room temperature for 1 h and analyzed by an infrared imaging scanner (Li‐Cor). Initial attempts to use β‐actin, GAPDH or HSC‐70 as a loading control for our Western analysis revealed consistent effects on their expression in the mitochondria by TILLIR (Figure 5d), while stained gels showed no significant difference in sample loadings, as such, we present the data without normalization.

The effect of TILLIR on phosphorylation of a protein was analyzed by normalizing the phosphor signal with total protein abundance using the same nitrocellulose membrane. To analyze the effect of TILLIR on AKT1‐S473‐P, we incubated a nitrocellulose membrane with a rabbit anti‐AKT1‐S473‐P antibody and mouse anti‐AKT antibody at the same time. The rabbit anti‐AKT1‐S473‐P and mouse anti‐AKT antibodies were detected with an Alexa‐680 anti‐rabbit secondary antibody and an Alexa‐800 anti‐mouse secondary antibody simultaneously. The phosphor signals from ERK1/2 and NDRG are weaker than the total ERK1/2 and NDRG protein signals. To analyze the effect of TILLIR on ERK1/2‐P and NDRG‐P, we first probed a nitrocellulose membrane with the rabbit anti ERK1/2‐P or NDRG‐P antibody. After we imaged the ERK1/2‐P or NDRG‐P signal with the Alexa‐680 anti‐rabbit secondary antibody at a high Intensity (high sensitivity), we then incubated the same membrane with the rabbit anti ERK1/2 or NDRG antibody and scanned the signals with the Alexa‐680 anti‐rabbit secondary antibody at a low intensity (low sensitivity). Because scanning at this low sensitivity would not be able to detect the weak signal from ERK1/2‐P or NDRG‐P, the detected signal was from the anti ERK1/2 or NDRG antibody. The rabbit antibodies against SGK1 (12103S), Tom20 (42406S), NDRG1‐T346‐P (5482S), NDRG (5196S), PRDX1 (8732S), ERK1/2‐P (9101S), ERK1/2 (9102S), AKT1‐S473‐P (4060S), and GAPDH (2118S) and mouse antibodies against β‐actin (3700S), HSP‐27 (2402S) and AKT (2920S) were purchased from Cell Signaling Technology. The rabbit antibodies against LC3 (14600‐1‐AP), p62 (18420‐1‐AP), PINK1 (23274‐1‐AP), PARK2 (14060‐1‐AP), and Tom70 (14528‐1‐AP) were bought from Proteintech. The rabbit MnSOD antibody (06‐984) was purchased from Millipore. The rat antibody against HSC70 (sc‐59560) was acquired from Santa Cruz Biotechnology.

### 2.10 qPCR

Total RNA was extracted with the ice‐cold RNAzol RT kit (Molecular Research Center) and quantified with NanoDrop 8000 (ThermoFisher). cDNAs were synthesized with the High‐Capacity cDNA Reverse Transcription Kit (Applied Biosystems, Part# 4368814). mRNAs were quantified with the Fast SYBR Green Master Mix (Applied Biosystems, Ref# 4385612) in Cycler 480 (Roche) and normalized to L32 mRNA. The primers used are listed in Table 1, and the fold difference in mRNA abundance between conditions (*F*) was calculated as described previously (Ferraris et al., 2002).

**TABLE 1:** List of primers

Gene names	Forward	Reverse
PGC−1α	GAGAGGCAGAAGCAGAAAGC	CTCAATTCTGTCCGCGTTGT
NDUFS1	GACCAGGGAGGTGAATGTGA	GTTCTTGTCCTCCACAGCAC
ATP5o	AGCTTCCTGAGTCCAAACCA	AAGAGCTCACACAGGTGACA
CYCS	GGCTGAGTCCTCTGGAAGAA	CGGCAATTCCAGGGCTTTAT
L32	ACCAGTCAGACCGATATGTG	ATTGTGGACCAGGAACTTGC

### 2.11 Statistical analysis

Data are expressed as mean ± standard error of the mean. In the analyses of mRNA and proteins, all readings were normalized to the result from the first mouse in the respective control group. Unpaired *t* tests and two‐way ANOVA were used as appropriate with GraphPad Prism 9.0.2. Tukey's post‐hoc testing was used for multiple comparisons in Two‐way ANOVA. *p *< 0.05 was considered significant.

## 3 RESULTS

### 3.1 TILLIR‐induced AKI is dependent on duration

**FIGURE 2:**
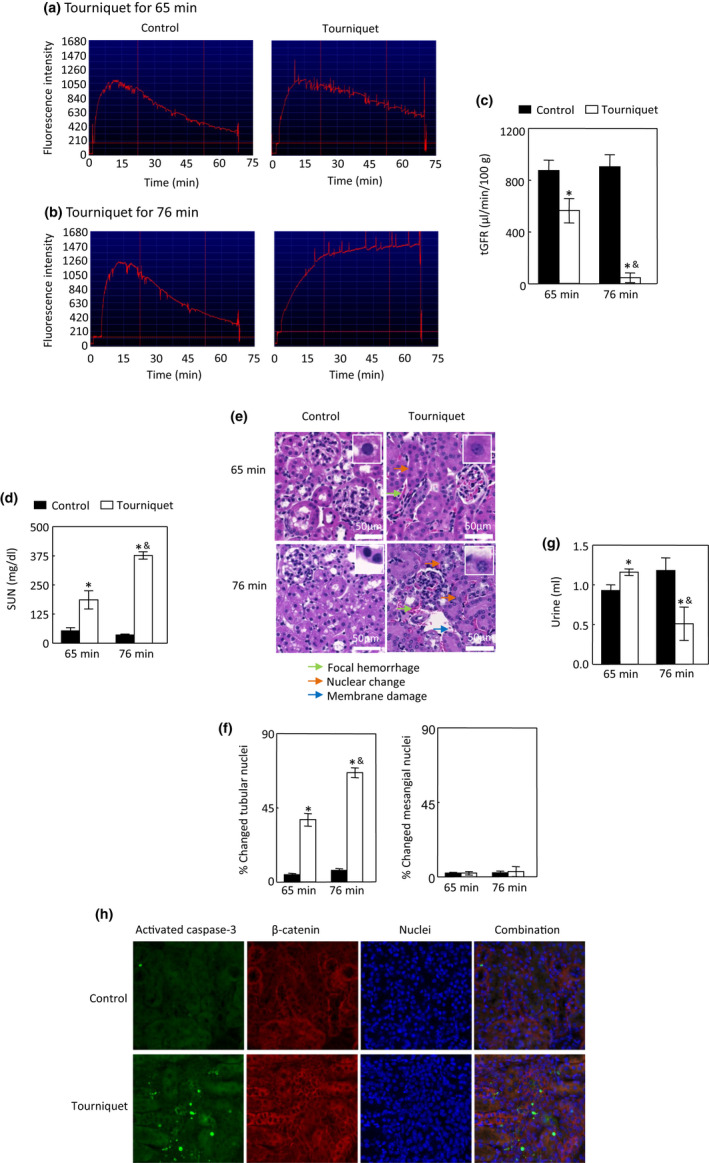
TILLIR induces AKI in a time‐dependent manner. Application of tourniquets for 65 min reduced tGFR (a–c), increased SUN levels (d) and induced peritubular focal hemorrhage and nuclear uneven staining in tubular cells, but not in mesangial cells (e, f). However, tourniquets for 65 min increased urinary output (g). Therefore, tourniquets for 65 min only induce mild AKI without oliguria. However, application of tourniquets for 76 min induced more reduction in tGFR (a–c), increases in SUN concentrations (d) and tubular nuclear changes than 65‐min tourniquet time and basolateral membrane damage, in addition to focal peritubular hemorrhage (e, f), and reduced urinary output (g). Thus, tourniquets for 76 min induce severe AKI. Tourniquets for 76 min activated caspase‐3 (h). *p < 0.05 versus respective control (unpaired *t* test), ^&^ *p*<0.05 versus 65 min tourniquet time (two‐way ANOVA). a and b: representative tGFR measurements, c: *n* = 6 for 65 min and *n* = 3 for 76 min, d: *n* = 5 for 65 min and *n *= 7 for 76 min, e: representatives of three independent experiments, f: *n* = 3, g: *n* = 5 for 65 min and *n* = 7 for 76 min. h: representatives of two independent experiments

Tourniquet application for 65 min significantly reduced tGFR and increased SUN levels (Figure 2a–d). 65 min of tourniquet application also resulted in focal peritubular hemorrhage and uneven nuclear staining in tubular cells, but not in mesangial cells (Figure 2e,f). However, this duration of tourniquet application significantly increased urinary output (28.2 ± 11.8%, *p *< 0.05, Figure 2g), indicating non‐oliguric AKI. In contrast, tourniquet application for 76 min induced a 91.9 ± 6.5% drop in tGFR (compared to only a 33.6 ± 9.8% drop in 65‐min tourniquet time) and a 1048.2 ± 149.0% increase in SUN concentrations (compared with only a 263.4 ± 82.4% increase in 65‐min tourniquet time (*p *< 0.05, Figure 2a–d). In addition to focal peritubular hemorrhage, tourniquets for 76 min resulted in more tubular cell nuclear changes (66.3 ± 3.0% vs. 37.7 ± 3.8% in 65 min, *p* < 0.05) and basolateral membrane damage (Figure 2e,f). Moreover, tourniquets for 76 min reduced urinary output by 62.3 ± 11.2% (Figure 2g). Application of tourniquets for 76 min was not significantly different in terms of pain scores compared with 65 min of tourniquet time. However, tourniquets for 76 min induced significantly less body weight loss compared with controls, likely due to lower urinary output (Table 2). We conclude that tourniquets for 76 min resulted in more severe AKI, and thus focused the remainder of our experiments on 76‐min tourniquet time. We found that tourniquets for 76 min activated caspase‐3 (Figure 2h).

**TABLE 2:** Application of tourniquet for 76 min reduces body weight loss

	Time (min)	Pain score	Body weight (g)		Difference	*p‐*value	*n*
			At beginning	At end			
Ischemia	0	0	23.3±0.7	21.1±0.7	2.1±0.5	>0.05	7
	65	3.6±0.8	23.3±0.5	21.6±0.6	1.6±0.5		
Ischemia	0	0	22.8±0.9	20.0±0.8	2.8±0.2	<0.005	6
	76	4.2±0.4	21.3±0.5	19.6±0.3	1.7±0.2		

Note

Mouse body weight was measured at the beginning and end of an experiment, respectively, unpaired *t* test.

### 3.2 TILLIR decreases mitochondrial function by reductions in mitochondrial Krebs cycle content and biogenesis

**FIGURE 3:**
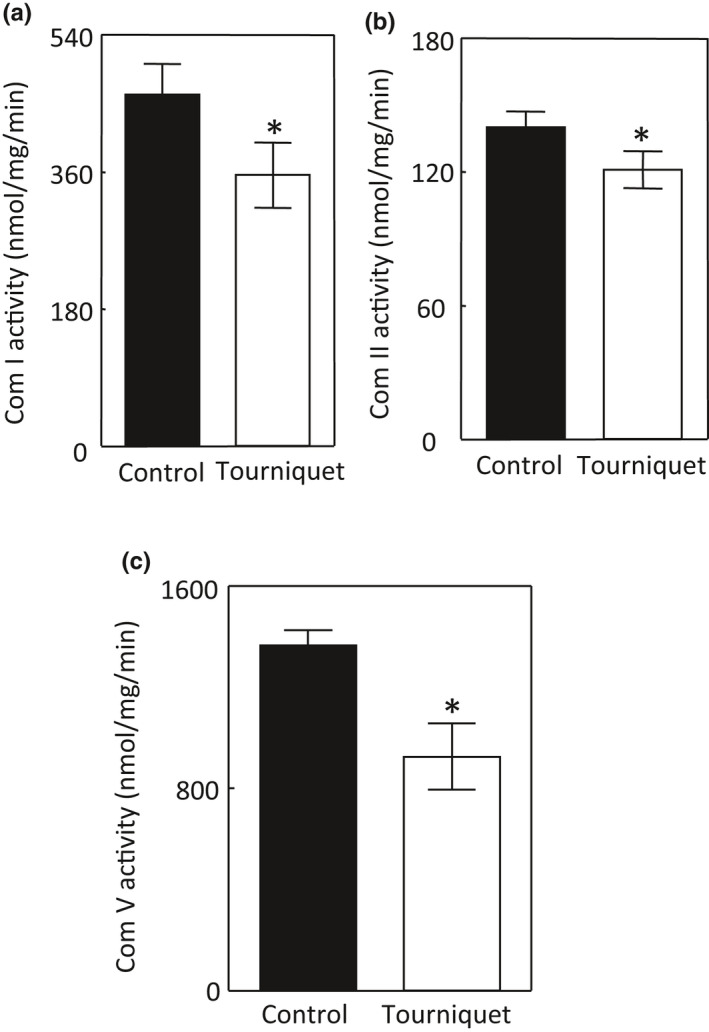
Tourniquets for 76 min reduce mitochondrial activities of complex I (a), complex II (b) and complex V (F0F1‐ATPase) (c). The complex I and II activities were assessed by colorimetric measurements of oxidation of 2, 6‐dichloroindophenol sodium. The complex V activity was measured colorimetrically with release of Pi. * *p *< 0.05 versus control (unpaired *t* test). a: *n* = 8, b: *n* = 13, and c: *n* = 6

**FIGURE 4:**
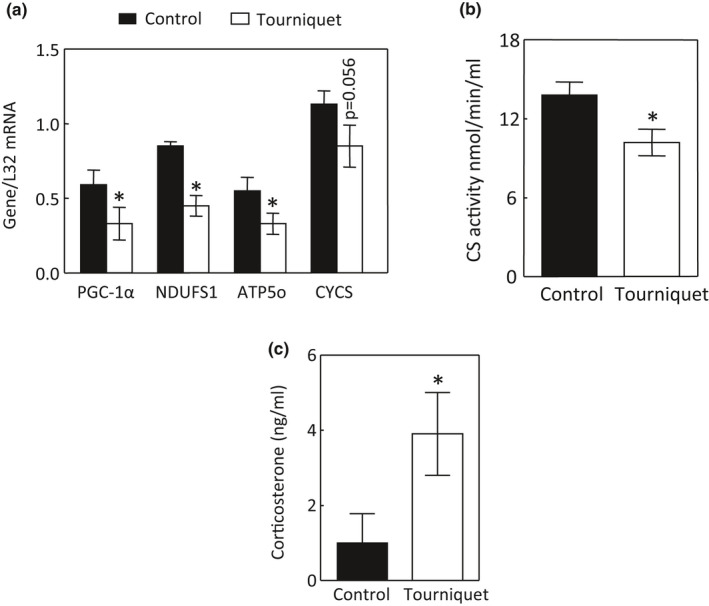
Tourniquets for 76 min reduce mitochondrial biogenesis in the renal cortex. Significant reductions in the mRNA levels of PGC‐1α, NDUFS1 and ATP5o (a) were found, as were TILLIR‐induced decrease in citrate synthase (CS) activity (b) and increase in serum corticosterone (c). *p < 0.05 versus respective control (unpaired *t* test). a: *n* = 7, b and c: *n* = 8

TILLIR significantly decreased mitochondrial complex I activity by 22.9 ± 3.0%, complex II activity by 16.2 ± 3.5%, and complex V activity by 33.3 ± 6.3% in the renal cortex (*p *< 0.05) (Figure 3a–c). TILLIR significantly decreased mRNA levels of PGC‐1α and its target genes NDUFS1 (NADH‐ubiquinone oxidoreductase 75 kDa subunit, the largest subunit of mitochondrial complex I), ATP5o (a subunit of the mitochondrial complex V) and possible CYCS that encodes cytochrome C (Figure 4a). In parallel, TILLIR also reduced mitochondrial citrate synthase activity by 23.3 ± 8.3%, an established marker of mitochondrial Krebs cycle content (Larsen et al., 2012) (Figure 4b). Because pathophysiologic levels of glucocorticoids suppress PGC‐1α expression (Annie et al., 2019; Rahnert et al., 2016), we measured serum levels of corticosterone, the major glucocorticoid in mice, and found that TILLIR increased corticosterone levels by 3.9 ± 1.4 fold (*p *< 0.05) (Figure 4c). We conclude that TILLIR decreases mitochondrial metabolic activity by reducing Krebs cycle content and mitochondrial biogenesis, which is associated with increased serum corticosterone.

### 3.3 TILLIR inhibits autophagy, but does not significantly affect PINK1‐PARK2 dependent mitophagy

**FIGURE 5:**
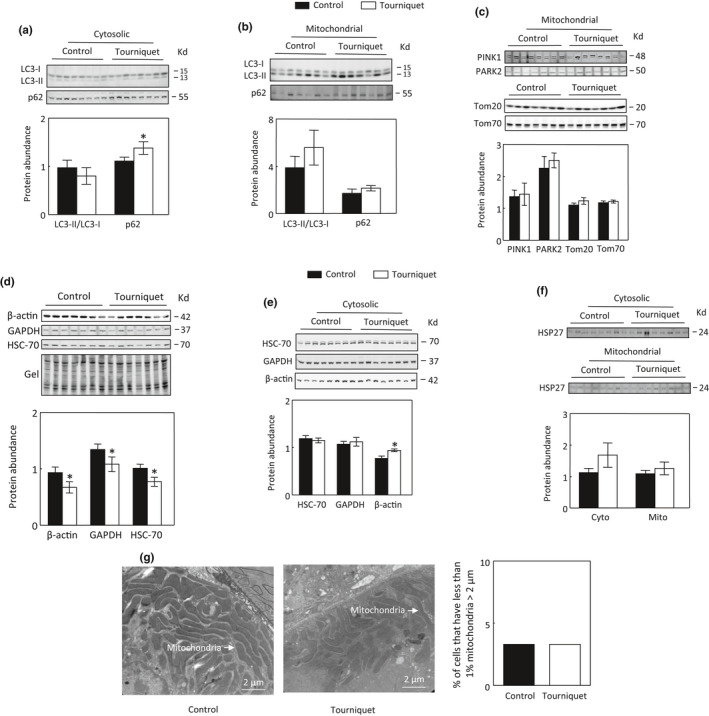
Tourniquets for 76 min inhibit autophagy, but not mitophagy in the renal cortex. TILLIR did not significantly affect the conversion of LC3‐I to LC3‐II in the cytosol and mitochondria and significantly increased p62 protein levels in the cytosol (a and b). TILLIR had no significant effect on mitochondrial protein abundance of PINK1, PARK2, Tom20, or Tom70 (C) but significantly decreased protein abundance of mitochondrial β‐actin, GAPDH and HSC‐70 (d). TILLIR had no significant effect on the protein abundance of cytosolic HSC‐70 or GAPDH but significantly increased the cytosolic level of β‐actin (e), and had no significant effect on the protein abundance of cytosolic or mitochondrial HSP27 (f) or percentages of cells that have less than 1% of mitochondria longer than 2 μm (g). *p < 0.05 versus respective control (unpaired *t* test). a: *n* = 8, b: *n* = 7, c: *n *= 7 or 8, d: *n* = 7, e and f: *n* = 8, g: *n* = 2

TILLIR did not affect the conversion of LC3‐I to LC3‐II in the cytosol or mitochondria but did increase protein abundance of p62 in the cytosol (Figure 5a,b). However, TILLIR did not significantly alter protein level of mitochondrial p62, suggesting that TILLIR did not affect mitophagy (Figure 5b). To examine this issue further, we measured the effect of TILLIR on protein abundance of mitochondrial PINK1, PARK2, Tom20, and Tom70 and found that none of them was significantly affected by TILLIR (Figure 5c). We initially attempted to use the mitochondrial β‐actin, GAPDH or HSC‐70 as a loading control for our Western analysis. However, TILLIR consistently decreased abundance of these proteins, while gel staining after transferring proteins onto membranes revealed no significant difference in sample loadings (5d). TILLIR had no significant effect on protein abundance of cytosolic HSC‐70, an important molecule in chaperone‐mediated autophagy (Dodson et al., 2013), or GAPDH, but increased cytosolic β‐actin (Figure 5e). TILLIR did not significantly affect cytosolic or mitochondrial HSP‐27 protein level (Figure 5e). To further determine whether TILLIR induced mitophagy, we performed a transmission microscopy study and found that TILLIR had no significant effect on the percentages of cells that had <1% of mitochondria longer than 2 μm (Figure 5g). We conclude that TILLIR inhibits autophagy but has no significant effect on PINK1‐PARK2‐mediated mitophagy.

### 3.4 TILLIR does not induce mitochondrial oxidative stress

**FIGURE 6:**
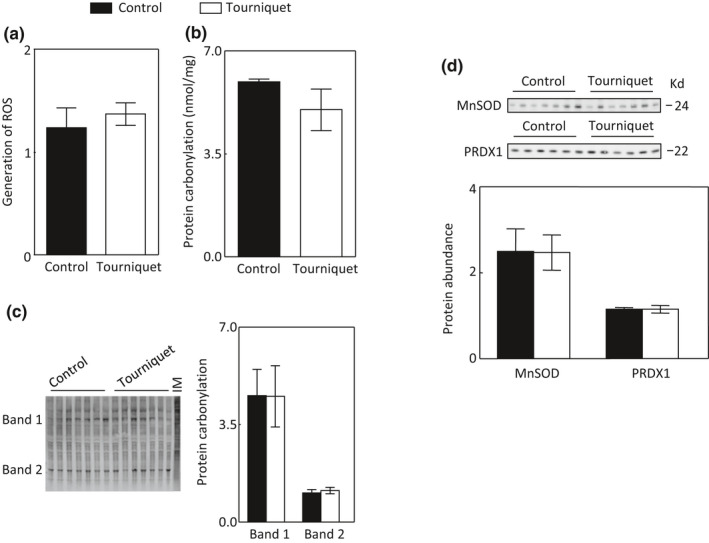
Application of tourniquets for 76 min has no significant effect on mitochondrial oxidative stress in the renal cortex. TILLIR did not significantly increase the mitochondrial ability to generate reactive oxygen species (ROS) (a), carbonylation of total mitochondrial proteins (b), or two major mitochondrial proteins (c). The carbonylation of mitochondrial proteins in the kidney inner medulla (IM) (Zhang et al., 2004) serves as a positive control (c). TILLIR also had no significant effect on the protein abundance of mitochondrial MnSOD or peroxiredoxin I (PRDX1) (d). a: *n* = 7, b: *n* = 8, c: *n* = 7 and d: *n* = 6 or 7

Mitochondrial oxidative stress regulates mitophagy (Dodson et al., 2013). As an additional test to determine whether mitophagy was involved in TILLIR‐induced AKI, we examined the effect of tourniquet on mitochondrial oxidative stress. TILLIR had no significant effect on the mitochondrial ability to generate reactive oxygen species or on total protein carbonylation (Figure 6a,b). To assess whether tourniquet might increase carboxylation of certain mitochondrial proteins, which may not be able to be detected by measuring the total protein carbonylation, we fractionated the mitochondrial proteins with gel electrophoresis and quantified two major bands. TILLIR did not significantly affect the carbonylation of these two bands either (Figure 6c). Moreover, TILLIR did not significantly affect levels of mitochondrial MnSOD, the first‐line defense against mitochondrial superoxide, or peroxiredoxin 1 (Prdx1), which metabolizes hydrogen peroxide and alkyl hydroperoxides (Figure 6d). We conclude that TILLIR does not induce mitochondrial oxidative stress in the kidney cortex.

### 3.5 TILLIR activates cytosolic and mitochondrial ERK1/2, mitochondrial AKT1 and SGK1

**FIGURE 7:**
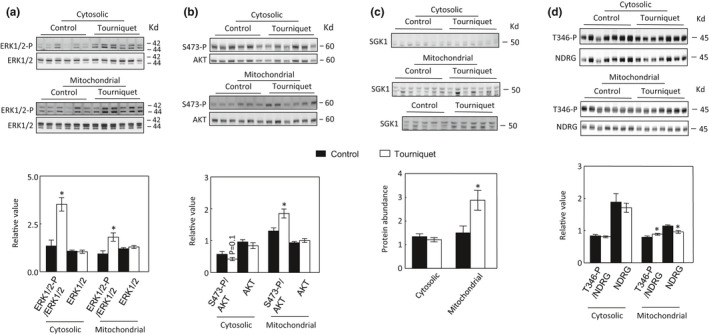
Application of tourniquets for 76 min significantly increases phosphorylation of ERK1/2 in both cytosolic and mitochondrial fractions (a) and phosphorylation of S473 of AKT1 only in the mitochondrial fraction (b) and has no significant effect on total protein abundance of ERK1/2 or AKT in either fraction (a & b). TILLIR only increases SGK1 protein abundance and activity in the mitochondrial fraction (c and d). The activity of SGK1 was measured by phosphorylation of T346 of NDRG, N‐myc downregulated gene (d). TILLIR reduces mitochondrial NDRG protein abundance (d). *p < 0.05 versus respective control (unpaired *t* test). a and b: *n* = 6, c: *n* = 7 or 12, d: *n* = 7

To gain mechanistic insights into the effects of TILLIR, we examined phosphorylation (activation) of ERK1/2, AKT1 and activity of SGK1 in the cytosolic and mitochondrial fractions. TILLIR significantly increased phosphorylation of ERK1/2 in both fractions (Figure 7a), whereas it only increased phosphorylation of S473 of AKT1 in the mitochondrial fraction (Figure 7b). TILLIR had no significant effect on total protein abundance of ERK1/2 or AKT in either fraction (Figure 7a,b). TILLIR increased SGK1 protein abundance and activity in the mitochondrial but not in the cytosolic fraction (Figure 7c,d). The SGK1 activity was measured by phosphorylation of NDRG, N‐myc downregulated gene, the protein known to be specifically phosphorylated by SGK1 (Murray et al., 2004) (Figure 7c,d). TILLIR also significantly decreased mitochondrial NDRG protein abundance (Figure 7d).

## 4 DISCUSSION

Mitochondrial oxidative stress has long been recognized as a major mechanism involved in renal I/R‐induced AKI (Nath & Agarwal, 2020). Ischemia dysregulates mitochondrial electron transport chain. The outburst of mitochondrial reactive oxygen species, mainly superoxide, was presumed to be the result of electron leaking at multiple non‐specific sites from a dysfunctional electron transport chain after reperfusion (Chouchani et al., 2014). However, Chouchani et al. (2014) identified a specific metabolic pathway in which superoxide is generated through reverse electron transport at complex I of the electron transport chain. Moreover, this process is shown to be driven by accumulation of the citric acid cycle metabolite succinate, which occurs during ischemia. Excessive production of mitochondrial reactive oxygen species damages mitochondria, triggering mitophagy as a defense mechanism to prevent further oxidative stress (Dodson et al., 2013; Wang, Cai, et al., 2020). Herein, TILLIR did not induce mitochondrial oxidative stress or PINK1‐PARK2‐dependent mitophagy (Figures 5 and 6). This is probably because the blood supply to the kidney was reduced and not restored due to blood volume depletion (De Rosa et al., 2018; Simon et al., 2018).

Because renal tubules absorb filtered glucose, amino acids, Na^+^ and other ions, the kidney is the second only to the heart in mitochondrial abundance and oxygen consumption at rest (Forbes, 2016). While TILLIR increases mitochondrial function 2 h after releasing tourniquets (Mansour et al., 2014), we found that TILLIR decreased mitochondrial function 22 h after release of tourniquets, revealing the importance of time after injury. We have found that decreases in mitochondrial function was mostly explained by reduction in mitochondrial content and biogenesis (Figures 3 and 4), which is a dynamic process that takes time to manifest. These data also illustrate that although the mitochondria may have similar size and number between control and AKI kidneys, they have different metabolic activities. Indeed, not every mitochondrion has the same content. For example, in the swine kidney, the cortical mitochondria have more enzymes involving beta oxidation, amino acid metabolism, and gluconeogenesis than the medullary mitochondria, which contain more tricarboxylic acid cycle enzymes and electron transport system proteins (Tuma et al., 2016). We observed that TILLIR markedly increased serum corticosterone levels (Figure 4c), which could play a critical role in mitochondrial mass because the synthetic glucocorticoid dexamethasone inhibits expression of PGC‐1α (Annie et al., 2019; Rahnert et al., 2016) and activates ERK1/2 (Kumar et al., 2009), which also reduces expression of PGC‐1α (Collier & Schnellmann, 2020).

Upon phosphorylation, AKT1 rapidly translocates from the cytosol to the mitochondria where it phosphorylates the β‐subunit of complex V (Bijur & Jope, 2003). In cultured rabbit primary proximal tubular cells, AKT activation increases complex I, III, and V activity (Shaik et al., 2008). Direct renal I/R increases phosphorylation of mitochondrial AKT1 within 60 min, followed by an increase of total AKT1 protein abundance. This effect has been interpreted as a defense mechanism against renal I/R‐induced injury (Lin et al., 2021). We have observed that TILLIR increased phosphorylation of AKT1 without significantly affecting the total AKT protein level in mitochondria 22 h after releasing tourniquets (Figure 7). We suggest that accumulation of activated AKT1 in mitochondria is a compensating mechanism for reduced mitochondrial function secondary to impaired mitochondrial biogenesis.

Renal I/R increases SGK1 transcript and protein levels (Rusai et al., 2009). Similar to the effect of dexamethasone in HEK293 cells (Hira et al., 2020), we found that TILLIR‐induced increases in SGK1 protein and activity were restricted to the mitochondria (Figure 7). Liver‐specific knockout of SGK1 markedly enhances hepatic autophagy (Zhou et al., 2019), indicating that SGK1 inhibits autophagy. SGK1 reduces mitochondrial reactive oxygen species (Aspernig et al., 2019), and inactivation of SGK1 increases mitochondrial reactive oxygen species and induces autophagy in *C*. *elegans* (Aspernig et al., 2019; Heimbucher et al., 2020). Although we observed that TILLIR increased the level and activity of mitochondrial SGK1, we detected no increase in mitochondrial reactive oxygen species (Figures 6 and 7). We did observe increase in cytosolic p62, however. Whether the activation of mitochondrial SGK1 contributes to TILLIR‐induced inhibition of autophagy remains to be determined.

One limitation of the current study is the associative nature of the findings and lack of a direct cause‐effect relationship. Reduced expression of PGC‐1α has been implicated in a variety of types of AKI (Annie et al., 2019; Collier & Schnellmann, 2020; Farahani et al., 2020; Fontecha‐Barriuso et al., 2019; Portilla et al., 2002; Rahnert et al., 2016; Ruiz‐Andres et al., 2016; Song et al., 2021; Tran et al., 2011). To address whether reduced expression of PGC‐1α contributes to or a consequence of TILLIR‐induced AKI, the current study needs to be repeated in mice that over express PGC‐1α in the renal tubules. Similarly, the effect of TILLIR on AKI needs to be examined under the condition of enhanced autophagy to determine whether reduced autophagy contributes to TILLIR‐induced AKI.

**FIGURE 8:**
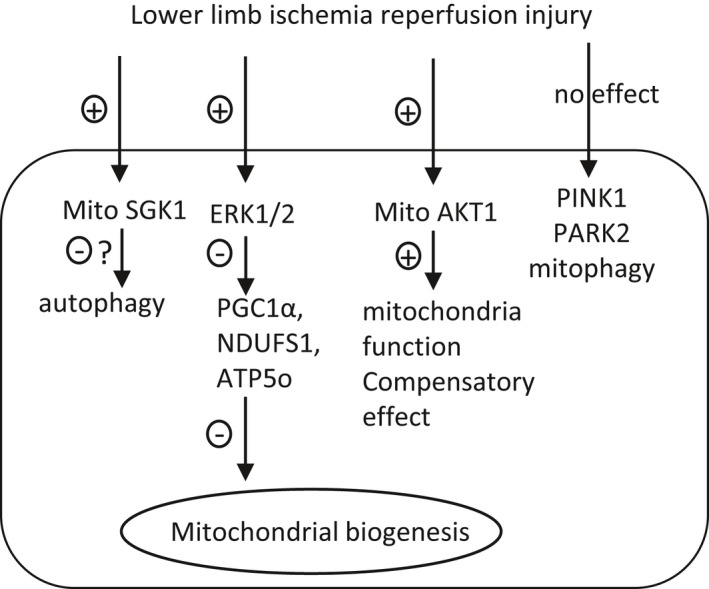
Proposed model to illustrate how TILLIR damages mitochondria in the injured kidney cortex. Mito mitochondria

In summary, TILLIR inhibits mitochondrial function in the renal cortex during AKI. This effect is associated with an increase in serum corticosterone, activation of cytosolic and mitochondrial ERK1/2, activation of mitochondrial AKT1, and decreases of mitochondrial content and biogenesis. TILLIR inhibits autophagy, possibly through activation of mitochondrial SGK1. In contrast, TILLIR has no significant effect on PINK1‐PARK2‐mediated mitophagy or mitochondrial oxidative stress. The present study suggests that reducing cellular demand for oxygen or enhancing autophagy in the kidney may be more efficacious in mitigating TILLIR‐induced AKI as opposed to targeting renal mitochondrial oxidative stress or mitophagy. Based on our present study and other investigators’ findings, we propose a model to illustrate how TILLIR affects mitochondria in the injured kidney cortex (Figure 8).

## DISCLAIMER

The content and views expressed in this article are the sole responsibility of the authors and do not necessarily reflect the views or policies of the Department of Defense or United States Government. Mention of trade names, commercial products, or organizations does not imply endorsement by the Department of Defense or US Government.

## CONFLICT OF INTEREST

Authors declare no conflict of interest.

## ETHICS STATEMENT

Authors declare no ethics violation.
